# Increased bone marrow uptake of 18F-FDG in leukemia patients: preliminary findings

**DOI:** 10.1186/s40064-015-1339-2

**Published:** 2015-09-17

**Authors:** Maya Kato Arimoto, Yuji Nakamoto, Koya Nakatani, Takayoshi Ishimori, Kouhei Yamashita, Akifumi Takaori-Kondo, Kaori Togashi

**Affiliations:** Department of Diagnostic Imaging and Nuclear Medicine, Kyoto University Graduate School of Medicine, 54, Shogoin Kawahara-cho, Sakyo-ku, Kyoto, Kyoto 606-8507 Japan; Department of Diagnostic Radiology, Kurashiki Central Hospital, 1-1-1 Miwa, Kurashiki, Okayama 710-0052 Japan; Department of Hematology and Oncology, Kyoto University Graduate School of Medicine, 54, Shogoin Kawahara-cho, Sakyo-ku, Kyoto, Kyoto 606-8507 Japan

**Keywords:** Leukemia, Bone marrow, 18F-FDG PET/CT

## Abstract

The aim of this retrospective study was to evaluate the characteristics of increased bone marrow uptake of 18F-FDG in patients with leukemia who underwent whole-body 18F-FDG PET/CT. The 18F-FDG PET/CT images of 9 patients with histologically proven leukemia were reviewed. The accumulation of 18F-FDG in the bone marrow was evaluated, and was compared with histological subtype, clinical course, and hematological findings. Nine patients (4 males, 5 females; age range, 5–58 years) had increased bone marrow uptake of 18F-FDG, including 6 patients with acute lymphoblastic leukemia, 1 with acute myeloid leukemia, 1 with chronic myeloid leukemia, and 1 with mature B cell neoplasm. Bone marrow uptake was generally diffuse but focal or inhomogeneous uptake was common, especially in the upper and lower extremities. Patients with increased bone marrow uptake of 18F-FDG commonly complained of fever and bone pain. No correlations between 18F-FDG uptake and peripheral blood findings were observed. Patients with leukemia may have increased bone marrow uptake of 18F-FDG on PET/CT, possibly reflecting leukemic cell activity. Leukemia can be included in the differential diagnosis when increased bone marrow uptake of 18F-FDG is observed.

## Background

Leukemia is a hematological malignancy characterized by malignant cells that exist mainly in the bone marrow (BM) and peripheral blood. An estimated 48,610 new cases of leukemia are diagnosed in the US every year, resulting in 23,720 deaths. Leukemia is the leading cause of cancer-related deaths in males aged <40 years, and the second leading cause of cancer-related deaths in females aged <20 years (Siegel et al. 2013).

Although 18F-fluorodeoxyglucose (FDG) positron emission tomography (PET) or 18F-FDG PET/computed tomography (CT) is widely used for the diagnosis, staging, and response assessment of various types of malignancy, it is not routinely used in patients with leukemia, because leukemia typically does not present with a solid tumor, and because clinical use of this investigation in patients with leukemia has only been described in few case reports (Takalkar et al. 2004; Rao et al. 2009; Takahashi et al. 2011; Su et al. 2013; Arslan et al. 2014; Parida et al. 2015). We previously reported a case of acute lymphoblastic leukemia associated with diffuse homogeneous BM uptake of 18F-FDG (Su et al. 2013), indicating that some patients with leukemia who have tumor cells in the BM aspirate also have increased BM uptake of 18F-FDG on PET/CT. Therefore, we hypothesized that increased BM uptake of 18F-FDG may be a characteristic finding in patients with leukemia.

Increased BM uptake of 18F-FDG is sometimes observed after chemotherapy, after administration of granulocyte colony-stimulating factor, in patients with malignant lymphoma, and in patients with multiple bone metastases. This increased uptake is considered to be caused by increased numbers or increased activity of normal hematopoietic cells or tumor cells in the BM. However, the frequency of increased BM uptake of 18F-FDG in patients with leukemia, and associations between uptake characteristics and laboratory findings, are currently unknown.

The purpose of this study was to evaluate the imaging characteristics of increased BM uptake of 18F-FDG in patients with leukemia.

## Patients and methods

### Patients

Patients were retrospectively identified from our clinical PET/CT database, in which all PET/CT studies in our institution have been consecutively registered. The diagnosis of each patient was confirmed by his or her electronic medical record after PET/CT study, because PET/CT is often performed before invasive procedures such as biopsy or surgery. Between December 2010 and November 2013, 50 18F-FDG PET/CT scans were performed at our institute in 33 patients with histologically proven leukemia. The exclusion criteria were as follows: (1) recent administration of chemotherapy within 30 days before PET/CT (n = 15), (2) recent stem-cell transplantation within 1 year before PET/CT (n = 3), (3) co-existing malignant or active inflammatory lesions (n = 2), (4) lymphoma type presentation of adult T-cell leukemia/lymphoma (n = 2), (5) chronic lymphocytic leukemia/small lymphocytic lymphoma (n = 1), (6) recent administration of granulocyte stimulation-factor (n = 0), and (7) insufficient follow-up data (n = 1). All but the last criteria are associated with increased bone marrow uptake (Su et al. 2013). After the exclusion of 24 patients, the imaging findings of the remaining 9 patients were analyzed (4 males, 5 females; age range, 5–58 years). 18F-FDG PET/CT was performed for pre-therapeutic staging in 5 patients (to evaluate potential extramedullary disease or an inflammatory process before stem cell transplantation or chemotherapy), for re-staging in 2 patients (relapse after a stable status lasting for at least 3 years), and to investigate prolonged unexplained pyrexia in 2 patients who were subsequently diagnosed with leukemia. Repeat PET/CT for response assessment after leukemia treatment was performed in 4 of the 9 patients and the images obtained before and after treatment were compared.

This retrospective study was approved by the ethics committee of our institute, and written informed consent for data access was obtained from all patients. For those younger than 20 years of age, written informed consent was obtained from both the parents and the patients.

## 18F-FDG PET/CT scanning

18F-FDG PET/CT was performed using a combined PET/CT scanner (Discovery ST Elite; GE Healthcare Ltd., Waukecha, WI USA). Patients fasted for at least 4 h before intravenous administration of 3.7 MBq/kg body weight of 18F-FDG. The 18F-FDG dose for children was determined according to the revised European Association of Nuclear Medicine pediatric dosage card. Approximately 1 h (range 46–96 min, mean 64 ± 15 min) after the administration of 18F-FDG, whole-body PET was performed, with 2–3 min per bed position. The scanning range was from the upper thigh to the skull in 3 patients, and was extended to include the lower extremities in the remaining 6 patients because they had increased uptake in the femurs or pain in the legs. The CT data were used for attenuation correction, and images were reconstructed using the 3-dimensional iterative reconstruction algorithm VUE Point Plus.

## 18F-FDG PET/CT image analysis

18F-FDG PET/CT images were visually interpreted by 2 board-certified nuclear medicine physicians (M.K.A., with 4 years of experience interpreting PET; and Y.N., with 18 years of experience interpreting PET) and the results were recorded by consensus. Increased BM uptake of 18F-FDG was defined as uptake that was greater than in the liver. This method is used to evaluate BM uptake in lymphoma (Carr et al. 1998, Cerci et al. 2014). The distribution and pattern of BM uptake were classified region by region as (a) diffuse and homogeneous or (b) focal or inhomogeneous. The most intensive foci within the BM were identified using a 3D-volume viewer (Advantage Workstation 4.4, GE, Little Chalfont, UK). The maximum standardized uptake value (SUV_max_) of the most intensive focus was measured manually. Care was taken to avoid organs with physiological uptake. The SUV_max_ of a region of interest located in the right lobe of the liver served as reference. Associated findings, such as splenomegaly, lymph node swelling, and extramedullary lesions, were also evaluated. The SUV_max_ of the BM was compared with the patient’s hematological findings.

## Results

The clinical characteristics, PET findings, and laboratory data of the patients are shown in Table 1. Increased BM FDG uptake was observed in all 9 patients; including a diffuse and homogeneous pattern throughout the body in 1 patient and the coexistence of diffuse/homogeneous and focal/inhomogeneous patterns in 8 patients.

**Table 1:** Clinical characteristics of patients with leukemia

Patient	1	2	3	4	5	6	7	8	9
Age (years)	35	5	24	10	6	58	24	16	8
Sex	F	F	M	F	F	F	M	M	M
Chief complaints									
Fever	+	+	−	+	+	+	+	+	+
Bone pain	+	+	+	+	+	+	+	−	+
Laboratory data									
WBC (/µL)	2700	6900	4400	18,100	11,900	1100	9100	175,100	11,100
Leukemic cells (%)	0	0	0	64	11.5	3	21.5	4.5	12
Hb (g/dL)	10.3	11.0	12.4	12.1	11.8	8.6	14.3	10.5	12.5
PLT (×10^3^/μL)	340	300	48	22	115	58	184	59	63
LDH (IU/L)	190	183	262	955	367	300	489	972	11,021
CRP (mg/dL)	5.1	4.2	7.3	2.4	11.9	8.3	3.2	0.6	0.8
FDG-PET									
Abnormal BM uptake	+	+	+	+	+	+	+	+	+
SUVmax of BM	6.8	4.5	5.6	6.0	6.1	6.1	14.0	4.8	5.9
SUVmax of liver	2.4	1.4	4.2	2.4	2.5	2.6	2.7	2.7	1.3
Lymph node swelling	−	−	−	−	−	−	−	−	−
Splenomegaly	−	−	−	+	+	−	+	+	+
Increased uptake in spleen	−	−	−	+	+	+	+	−	+
Extramedullary lesion	−	−	−	−	−	−	−	−	−
Diagnosis	B-ALL	B-ALL	B-ALL	B-ALL	B-ALL	AML	B-ALL	CML	mature B
Time to diagnosis (months)	20	4	6	0	1	0	0	0	1
Treatment	CBT	CTX	CBT	CTX	CTX	BMT	CBT	CTX	CTX
Outcome	CR	CR	PR	PR	PR	PR	PR	Dead	–
Follow-up (months)	33	22	21	19	18	17	14	1	3

*WBC* white blood cell count, *Hb* hemoglobin, *PLT* platelet count, *LDH* lactate dehydrogenase, *CRP* C-reactive protein, *BM* bone marrow, *B-ALL* B-cell acute lymphoblastic leukemia, *AML* acute myeloid leukemia, *CML* chronic myeloid leukemia, *CBT* cord blood stem cell transplantation, *CTX* chemotherapy, *BMT* bone marrow transplantation, *CR* complete remission, *PR* partial remission

The BM SUV_max_ of the 9 patients ranged from 4.5 to 14.0 (mean: 6.6 ± 2.8) whereas the SUV_max_ of the liver ranged from 1.3 to 4.2 (mean: 2.5 ± 0.8). All 9 patients underwent BM biopsy within 8 days (mean 4.3 days) of PET/CT. The findings were confirmed histopathologically in 8 patients. In the remaining patient (Patient 9), the results were inconclusive despite repeat BM examinations; instead, leukemia was confirmed based on the presence of leukemic cells in the peripheral blood. The final diagnosis was acute lymphoblastic leukemia in 6 patients, acute myeloid leukemia in 1 patient, chronic myeloid leukemia in 1 patient, and mature B-cell neoplasm in 1 patient.

**Fig. 1:**
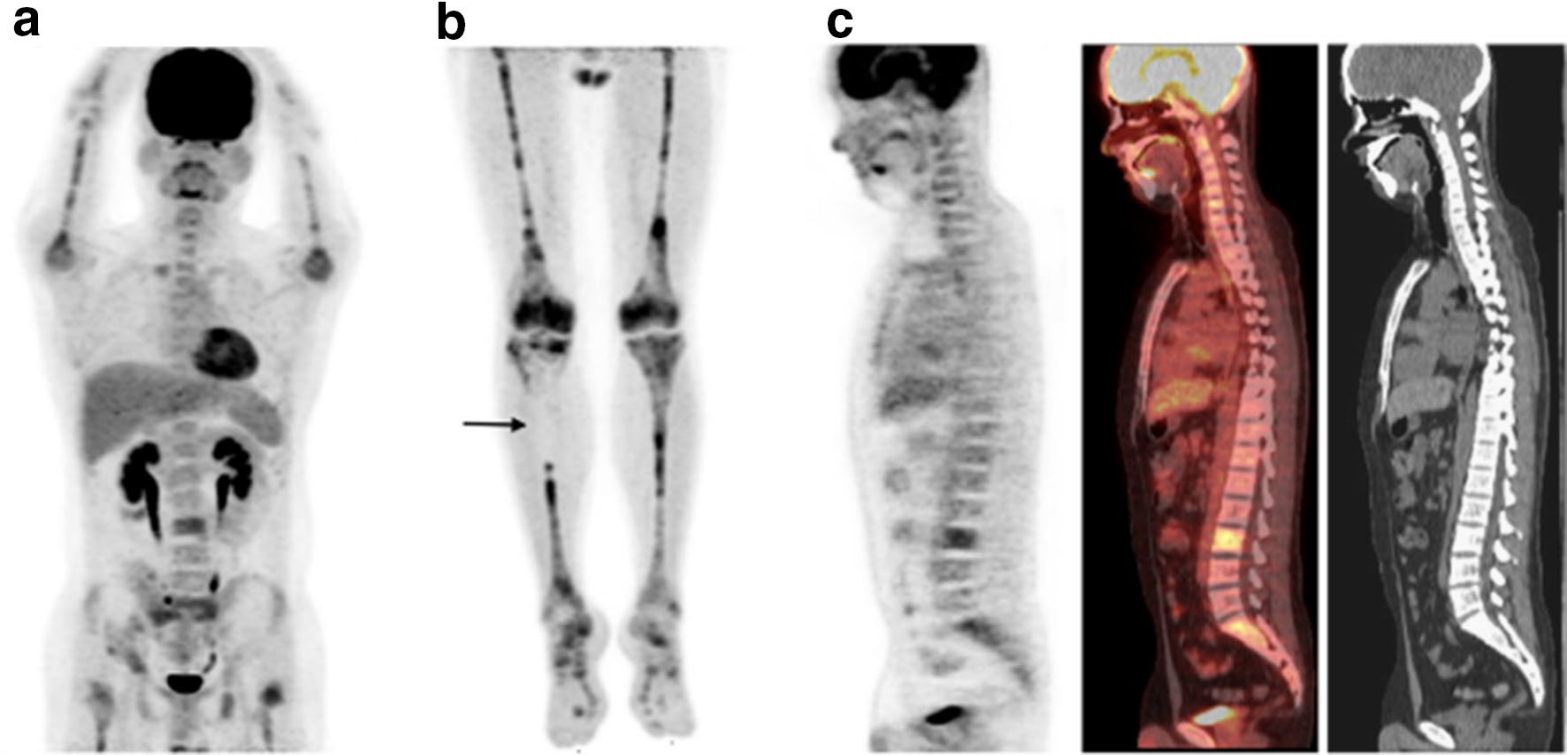
18F-FDG PET/CT images of a 24-year-old man with B-cell acute lymphoblastic leukemia (Patient 3). Maximum-intensity projection PET image extended to the legs (**a**, **b**) and sagittal PET/CT images (**c**), showing inhomogeneous bone marrow uptake. There are areas of decreased 18F-FDG uptake in the legs, especially in the proximal right tibia (**b**, *arrow*)

The distribution and pattern of BM uptake of 18F-FDG are shown in Table 2. The BM biopsy site confirmed to contain leukemic cells and the site where the BM SUV_max_ was recorded are also indicated in Table 2. The pattern of increased 18F-FDG uptake was generally diffuse, with frequent focal or inhomogeneous uptake, especially in the upper and lower extremities (Fig. 1).

**Table 2:** Patterns of Abnormal Bone Marrow Uptake of 18F-FDG

Patient	Skull	C	T	L	Pelvis	Sternum	Rib	Humerus	Radius/ulna	Femur	Tibia/fibula
1	–	D	D	D	D^a^	D	D	D	D	D^b^	D
2	–	–	–	–	–	D	–	D	D	F	D^a,b^
3	–	–	–	D	D^a^	–	–	F	NA	F^b^	F
4	–	D	D	D	D^a^	D	–	F^b^	–	D	D
5	–	D	D	D	D^a^	D	–	F	D	F^b^	D
6	–	D	D	D^b^	D^a^	D	D	F	–	F	–
7	–	D	D	D	D^a,b^	D	D	F	–	F	NA
8	–	D	D	D	D^a,b^	D	–	D	–	F	F
9	D	D	D	D	D	D	D	D	D	D^b^	F

*C* cervical vertebra, *T* thoracic vertebra, *L* lumbar vertebra, *D* diffuse and homogeneous increased uptake, *F* focal or inhomogeneous increased uptake, – no increased uptake, *NA* not available (out of scan)

^a^The bone marrow biopsy site was confirmed to contain leukemic cells

^b^The most intensive foci of each patient, in which SUV_max_ was measured

All 9 patients complained of fever or bone pain. In the 2 patients with fever of unknown origin, the hypermetabolic activity of the BM was useful in the determination of the optimal biopsy site, and resulted in the accurate diagnosis of leukemia.

After PET/CT and obtaining the final diagnosis, all patients received chemotherapy, followed by BM transplantation in 4 patients (Patients 1, 3, 6, and 7). Four patients (Patients 2, 3, 4, and 5) underwent repeat PET/CT after treatment. A decrease in the BM uptake of 18F-FDG following treatment was determined in these four patients based on comparisons with the pre-treatment PET/CT images. In addition, repeat BM examinations confirmed the decrease in number of leukemic cells. The outcome of the other patients varied, as shown in Table 1.

**Fig. 2:**
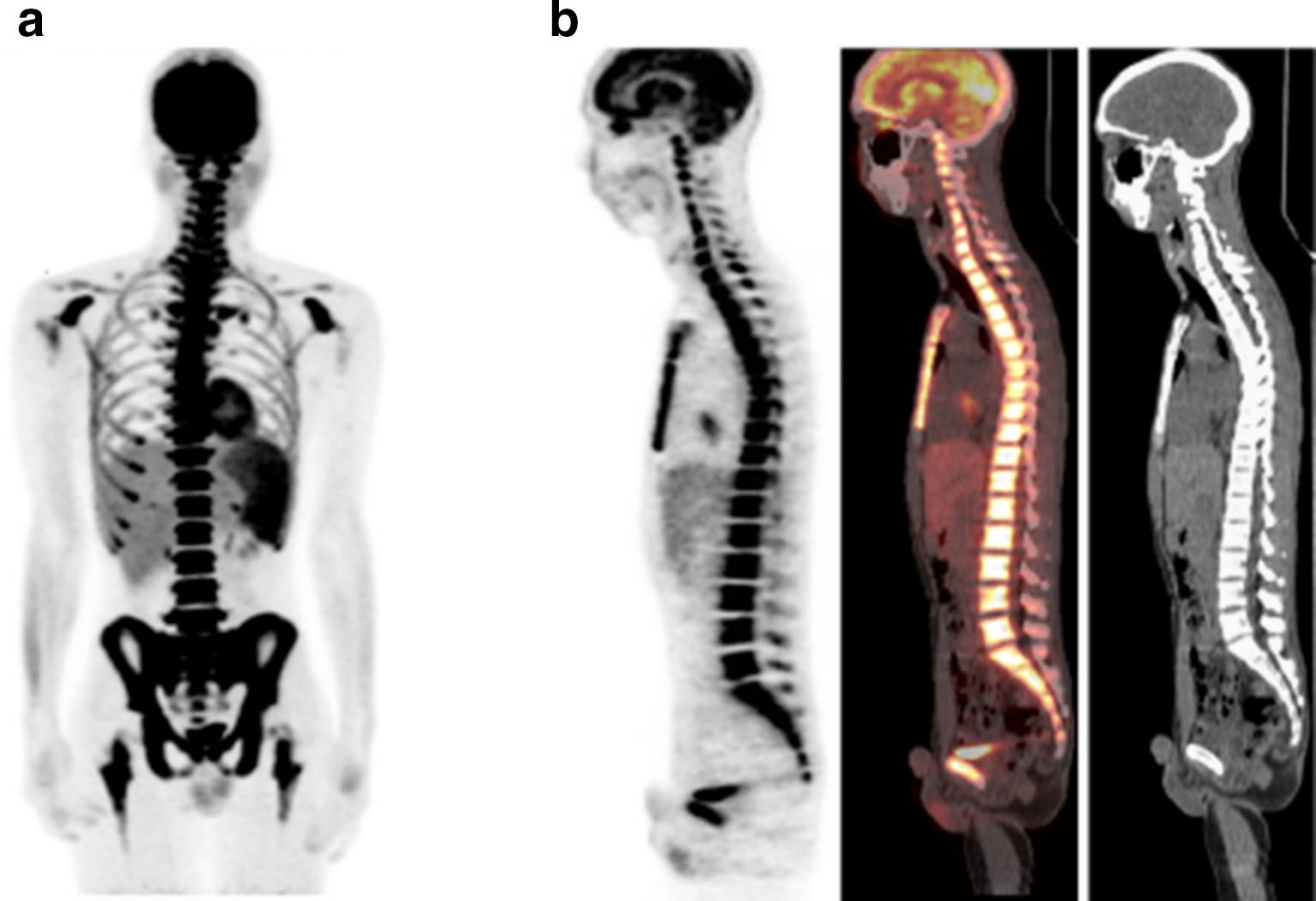
18F-FDG PET/CT images of a 24-year-old man with B-cell acute lymphoblastic leukemia (Patient 7). Maximum-intensity projection PET image (**a**) and sagittal PET/CT images (**b**), showing diffuse homogeneous bone marrow uptake in the body trunk and focal uptake in the extremities. The white blood cell count was elevated (9100/μL) and leukemia was confirmed by the detection of leukemic cells in the peripheral blood (21.5 %)

18F-FDG uptake was increased in the spleen of 5 patients (Fig. 2). None of the patients had increased uptake in the lymph nodes or in extramedullary sites. No associations were observed in this small population between SUV_max_ of the BM and the patients’ hematological data such as the white blood cell count, hemoglobin level, hematocrit, platelet count, C-reactive protein level, and lactate dehydrogenase level.

## Discussion

In this study, all 9 patients with leukemia had increased BM uptake of 18F-FDG on PET/CT. In 8 of them, leukemic cells in the BM were confirmed by BM examination performed at the same time. In the 4 patients who underwent repeat 18F-FDG PET/CT after chemotherapy, the decrease in the abnormal uptake was consistent with the decrease in the number of leukemic cells determined on repeat BM examination. These results suggest that an increase in BM uptake reflects an increase in either the number or the metabolic activity of leukemic cells in the BM, although normal hematopoietic cells may also demonstrate hyperactivity. Carr et al. (1998) reported that visual interpretation of BM uptake of 18F-FDG on whole-body PET could accurately identify BM infiltration by lymphoma. Some types of leukemia and lymphoma are histologically and immunophenotypically similar, and represent different clinical presentations of the same neoplasm (Sato et al. 2008; Vardiman et al. 2009). It is therefore reasonable that leukemic cells may cause increased BM uptake of 18F-FDG.

In our patients, increased BM uptake of 18F-FDG was generally observed in the vertebrae, pelvis, sternum, ribs, and extremities. Although the high BM uptake is not specific for leukemic infiltration since reactive changes caused by anemia or use of colony stimulating factors may show similar findings, the distribution of increased uptake was greater than the distribution of physiological uptake in red BM, which might be characteristic of, but not limited to leukemia.

Some of the patients in this study had focal or inhomogeneous BM uptake of 18F-FDG, especially in the extremities. This may reflect either the localization of leukemic cells in the BM or focal areas with a decrease in the number or activity of leukemic cells because of BM necrosis, which is caused by hypoxemia after the failure of the microcirculation. In patients with leukemia, the leukemic population may outgrow its blood supply and impinge upon vascular structures (Janssens et al. 2000). BM necrosis at sites without increased FDG uptake was not confirmed histopathologically in our patients because of clinical and ethical considerations.

Use of PET to detect focal bone localization of leukemia has been reported in several cases, especially in patients with relapse (Endo et al. 2004; von Falk et al. 2009; Ciarallo et al. 2011). In 1 patient in this study (Patient 2), BM aspiration from an area of increased uptake in the tibia resulted in definitive diagnosis of leukemia. 18F-FDG PET/CT may be useful for guiding the site of BM aspiration in such cases.

18F-FDG PET/CT has also been used in response assessment after leukemia treatment (Nakajo et al. 2007). In our study, the decrease in BM uptake after therapy coincided with a decrease in the number of leukemic cells in the BM in 4 patients. Therefore, 18F-FDG PET/CT performed after treatment may be useful for non-invasive response assessment in patients with increased pre-treatment BM uptake of 18F-FDG. However, whether a reactive increase in the BM uptake of 18F-FDG after treatment will adversely affect the diagnostic utility of this approach remains to be determined.

18F-FDG PET/CT may enable early diagnosis of leukemia in patients without leukemic cells in the peripheral blood. It is likely to be especially useful in the small proportion of patients with relatively normal blood cell counts at the initial presentation (Kato et al. 2011). If leukemic cells exist only in the BM, BM aspiration or biopsy is necessary to confirm the diagnosis. As bone pain and fever are non-specific symptoms, diagnosis may be delayed in patients presenting with these symptoms (van del Have et al. 2012; Parida et al. 2015). In this study, the time from the detection of the first symptoms to the diagnosis was longer in the 3 patients without than in the 6 patients with leukemic cells in the peripheral blood. In such cases, imaging modalities such as magnetic resonance (Bartakke et al. 2007; Kato et al. 2011) and PET/CT (Ennishi et al. 2009; Su et al. 2013; Arslan et al. 2014) may enable the earlier detection of BM abnormalities and thus an earlier initiation of treatment (Derlin et al. 2011). In fact, PET/CT is of greater utility than MRI because it can easily scan the whole body.

This study had several limitations. As a retrospective study, there may have been a substantial selection bias based on the decisions of the physicians to request 18F-FDG PET/CT. In addition, because of the small size of the study population, relationships between leukemia types and BM uptake patterns could not be fully investigated. Leukemia was not histopathologically confirmed at all sites with increased BM uptake because of clinical and ethical considerations. Accordingly, it cannot be ruled out that the increased BM uptake did not always reflect the presence of leukemic cells. The correlation between the level of leukemic cell infiltration and SUV_max_ was not determined, because data on the infiltration rate were only available for only 3 of 9 patients. However, taken together, the available BM examination results and the clinical follow-up findings strongly suggest that the BM uptake of 18F-FDG reflects leukemic cells activity. Further prospective studies with larger numbers of patients are warranted to assess the clinical utility of 18F-FDG PET/CT in patients with leukemia.

## Conclusions

Increased BM uptake of 18F-FDG can be observed in patients with leukemia. 18F-FDG PET/CT may be useful for the non-invasive assessment of patients with suspected leukemia, determination of optimal biopsy sites, and non-invasive response assessment. Leukemia can be included in the differential diagnosis of patients with increased BM uptake of 18F-FDG on PET/CT.
